# The influence of demographic characteristics on constipation symptoms: a detailed overview

**DOI:** 10.1186/s12876-020-01306-y

**Published:** 2020-06-03

**Authors:** Sanne J. Verkuijl, Rob J. Meinds, Monika Trzpis, Paul M. A. Broens

**Affiliations:** 1 Department of Surgery, Anorectal Physiology Laboratory, University of Groningen, University Medical Center Groningen, Hanzeplein 1, P.O. Box 30 001, 9700 RB Groningen, the Netherlands; 2 Department of Surgery, Division of Pediatric Surgery, University of Groningen, University Medical Center Groningen, Groningen, the Netherlands; 3 Department of Gastroenterology and Hepatology, Medisch Spectrum Twente, Enschede, the Netherlands

**Keywords:** Constipation, Digestive symptoms, Demographic factors, Diagnostic procedure

## Abstract

**Background:**

Diagnosing constipation remains difficult and its treatment continues to be ineffective. The reason may be that the symptom patterns of constipation differ in different demographic groups. We aimed to determine the pattern of constipation symptoms in different demographic groups and to define the symptoms that best indicate constipation.

**Methods:**

In this cross-sectional study the Groningen Defecation and Fecal Continence questionnaire was completed by a representative sample of the adult Dutch population (*N* = 892). We diagnosed constipation according to the Rome IV criteria for constipation.

**Results:**

The Rome criteria were fulfilled by 15.6% of the study group and we found the highest prevalence of constipation in women and young adults (19.7 and 23.5%, respectively). Symptom patterns differed significantly between constipated respondents of various ages, while we did not observe sex-based differences. Finally, we found a range of constipation symptoms, not included in the Rome IV criteria, that showed marked differences in prevalence between constipated and non-constipated individuals, especially failure to defecate (∆ = 41.2%).

**Conclusions:**

Primarily, we found that certain symptoms of constipation are age-dependent. Moreover, we emphasize that symptoms of constipation not included in the Rome IV criteria, such as daily failure to defecate and an average duration of straining of more than five minutes, are also reliable indicators of constipation. Therefore, we encourage clinicians to adopt a more comprehensive approach to diagnosing constipation.

## Background

Constipation is a common gastrointestinal disorder, with prevalences varying between 2.4 and 30.7% [1–9]. In addition, it is known that certain demographic groups, such as women and the elderly, are more prone to constipation [1–3]. The relation between level of education, living in an urban or rural environment, and/or body mass index (BMI) and the prevalence of constipation has also been studied, but with contradictory results [1–4, 10–12]. Despite constipation being a common disorder, it remains difficult to diagnose it and its treatment is often ineffective [13]. The difficulty with diagnosing constipation could be the fact that constipated individuals present a range of clinical symptoms that are difficult to define objectively [14, 15]. Furthermore, making a correct diagnose could be influenced by the fact that deviating stool frequency and deviating consistency are often considered the most straightforward symptoms of constipation, despite reports that other constipation symptoms, for example incomplete defecation, may predominate in constipated individuals [13, 16].

The Rome IV criteria were developed in an attempt to improve the diagnosis of constipation and are commonly used in clinical practice and research [17]. These criteria combine objective symptoms, such as stool frequency with subjective symptoms, such as sensation of anorectal obstruction [17–20]. Although the Rome IV criteria cover the most important symptoms of constipation, additional symptoms exist in constipated individuals. The symptoms we often encounter in clinical practice are, for example, anal pain or re-defecation within 1 h of stool passage. It is important to create awareness among clinicians of the relevance of these symptoms, because it would facilitate a more inclusive and effective approach to the diagnosis of constipation.

With these considerations in mind, our primarily aim was to determine the specific patterns of constipation symptoms in different demographic groups in a large population. We examined whether a more individual diagnostic approach could be achieved. Secondly, we aimed to define the symptoms that best indicate functional constipation by investigating a wide range of constipation symptoms.

## Methods

### Study design

We performed a cross-sectional study of the adult Dutch population between 1 September 2015 and 1 November 2015, using the validated Groningen Defecation and Fecal Continence questionnaire [21]. From the 3031 respondents who started filling out the questionnaire, 1642 (54.2%) filled it out completely. Survey Sampling International in Rotterdam, the Netherlands, an agency that specializes in conducting surveys, randomly selected a representative cohort of 1259 respondents from the completed questionnaires. This cohort was based on the population pyramid of the Netherlands according to sex, age, level of education and region, as reported by Statistics Netherlands [22]. In order to avoid possible bias, we excluded respondents who either had a medical history involving bowel functioning or who used medication that could influence the bowel system. As regards medical history, we excluded 250 (19.9%) respondents who either reported having a history of bowel surgery (intestinal resection, perianal fistula operation, anal sphincter operation, hemorrhoid operation, prostate operation) or who suffered from somatic diseases that could influence their bowels and anorectal functioning, such as rectal prolapse, inflammatory bowel diseases, diabetes, cerebral stroke, neurological disorders (spinal cord injury, multiple sclerosis), or congenital disorders (anorectal malformation, Hirschsprung’s disease, sacrococcygeal teratoma, or spina bifida). Additionally, we excluded another 117 (9.3%) respondents who reported using medication known to have constipation as a side-effect of more than 1 %, as reported by the Netherlands Pharmacovigilance Centre, Lareb [23]. These medicines included certain opiates, sympathomimetics, calcium channel blockers, and antipsychotics. Altogether we excluded 367 (29.2%) respondents on the basis of their medical history and/or medication use.

### Assessment of demographic variables

We divided the respondents into different demographic subgroups on the basis of their sex, age, level of education, living in an urban or rural environment, and BMI. Three age groups were formed based on respondents’ age percentiles: 18 to 38-year-olds, 39 to 54-year-olds, and 55 to 80-year-olds. Respondents’ highest level of education was classified as either tertiary (university or college), secondary (high school or vocational education), or primary (primary or middle school). The division between an urban or rural living environment was determined according to whether respondents reported living in a village or in a town or city. Based on respondents’ reported length and weight, we classified their BMI (kg/m^2^) as either underweight (< 18.5 kg/m^2^), normal weight (18.5 to 25 kg/m^2^), overweight (25 to 30 kg/m^2^), or obese (> 30 kg/m^2^). Female respondents were additionally required to provide information on their obstetric history by answering detailed questions regarding the number of childbirths, ways of delivery, duration of vaginal delivery, and possible difficulties that occurred during vaginal delivery.

### Assessment of constipation complaints

We diagnosed constipation in accordance with the Rome IV criteria for functional constipation that included straining, lumpy or hard stools (Bristol stool form type 1 or 2), incomplete evacuation, anorectal blockage, manual maneuvers to facilitate defecation, and reduced stool frequency (less than three bowel movements per week, which was assessed by asking the respondents “On average, how often do you empty your bowels?”) [17]. In order to meet the criteria for constipation the respondents had to suffer from at least two of the above complaints, plus rarely having loose stools without prior use of laxatives. Furthermore, we enquired about additional symptoms of constipation that we often encountered in clinical practice: failure to defecate, duration of straining, abdominal bloating, anal pain, abdominal pain, and re-defecation within 1 h of stool passage. In accordance with the Rome IV criteria, respondents with abdominal pain and/or bloating were not excluded from being constipated [17].

### Statistical analysis

Data were analyzed with SPSS for Windows, Version 23.0 (IBM SPSS Statistics, IBM Corporation, Armonk, NY). Proportions were reported as prevalence percentages with corresponding 95% confidence intervals (CI), which were compared using Pearson’s chi-square test. Differences in prevalences between certain groups were reported as delta prevalences (∆). Univariate and multivariate binary logistic regression models were used to test the association of demographic characteristics with the likelihood of constipation, for which all assumptions and interactions were checked. Logistic regression results were reported as odds ratio (OR) with corresponding 95% CI. Variables that tended towards significance (*p* < 0.10) in the univariate analyses were included in the multivariate model. Stata 14 (StataCorp, College Station, TX) was used for spline regression analysis of the relationship between age, sex, and the probability of functional constipation. Finally, two-sided *p* values of less than 0.05 were considered statistically significant.

## Results

### Respondent characteristics

We included 892 respondents in this study of whom 405 (45.4%) were male (Table 1). The median age of the respondents was 47 years and ranged from 18 and to 80 years. The majority of the respondents had either a secondary (40.9%) or tertiary (39.9%) level of education. Living in an urban environment was more common than living in a rural area (64.3 and 35.7% respectively). Almost half (47.2%) of the respondents had a normal weight according to the BMI, while 2.7% were underweight, 32.3% overweight, and 17.8% obese. Of all respondents, 247 (27.7%) drank less than 1,5 L water on a daily basis.

**Table 1:** Respondent characteristics

Demographic features	n (%)
**Overall**	892 (100.0)
**Sex**	
Men	405 (45.4)
Women	487 (54.6)
**Age groups**	
18–38 years	298 (33.4)
39–54 years	300 (33.6)
55–80 years	294 (33.0)
**Educational level**	
Primary	171 (19.2)
Secondary	365 (40.9)
Tertiary	356 (39.9)
**Living environment**	
Rural	318 (35.7)
Urban	574 (64.3)
**Body mass index**	
Underweight	24 (2.7)
Normal weight	421 (47.2)
Overweight	288 (32.3)
Obese	159 (17.8)
**Dietary factors**	
Water intake < 1.5 L/day	247 (27.7)
Vegetables < 3 spoons/day	223 (25.0)
Fruits < 2 pieces/day	473 (53.0)
Whole grain bread < 3 slices/day	288 (32.3)

### Prevalence and likelihood of constipation in different demographic groups

The Rome IV criteria for functional constipation were fulfilled by 15.6% of the respondents. Subsequently, we analyzed the prevalence and the likelihood of constipation separately for different demographic groups (Table 2). We found that women suffered from constipation significantly more often than men (19.7% versus 10.6%, *p* < 0.001). Moreover, there was a significant difference in the prevalence of constipation between age groups (*p* < 0.001), whereby prevalence was highest in the youngest age group (23.5%, Table 2). Respondents who drank less than 1.5 L water and ate less than 3 spoons vegetables per day, had a significant higher prevalence of constipation, compared to the ones with more fluid and vegetable intake. We found no significant difference between the prevalence of constipation in respondents with different levels of education, living environments, or BMI classifications (Table 2).

**Table 2:** The prevalence and likelihood of constipation in different demographic groups and dietary factors

Prevalence of constipation				Likelihood of constipation			
				Univariate logistic regression		Multivariate logistic regression	
**Demographic features**	%	95% CI	*p* value	Odds ratio (95% CI)	*p* value	Odds ratio (95% CI)	*p* value
**Overall**	15.6	13.2–18.0					
**Sex**			< 0.001**				
Men	10.6	7.6–13.6		Reference		Reference	
Women	19.7	16.2–23.3		2.07 (1.40–3.04)	< 0.001**	2.08 (1.39–3.10)	< 0.001**
**Age groups**			< 0.001**				
18–38 years	23.5	18.6–28.3		Reference		Reference	
39–54 years	13.3	9.5–17.2		0.50 (0.33–0.77)	0.002**	0.49 (0.31–0.77)	0.002**
55–80 years	9.9	6.4–13.3		0.36 (0.22–0.57)	< 0.001**	0.38 (0.23–0.64)	< 0.001**
**Educational level**			*0.13*				
Primary	11.7	6.8–16.6		Reference		Reference	
Secondary	14.8	11.1–18.5		1.31 (0.76–2.27)	*0.33*	1.32 (0.74–2.37)	*0.35*
Tertiary	18.3	14.2–22.3		1.69 (0.98–2.89)	*0.06*	1.63 (0.90–2.94)	*0.11*
**Living environment**			*0.21*				
Rural	13.5	9.7–17.3		Reference			
Urban	16.7	13.7–19.8		1.28 (0.87–1.90)	*0.21*		
**Body mass index**			*0.21*				
Underweight	12.5	−1.8–26.8		0.70 (0.21–2.43)	*0.58*	0.58 (0.16–2.04)	*0.40*
Normal weight	16.9	13.3–20.5		Reference		Reference	
Overweight	12.2	8.4–15.9		0.68 (0.44–1.06)	*0.09*	0.96 (0.60–1.52)	*0.85*
Obese	18.9	12.7–25.0		1.15 (0.72–1.84)	*0.57*	1.72 (1.02–2.90)	0.04*
**Dietary factors**							
Water intake < 1.5 L/day	21.5	16.3–26.6	0.003**^a^	1.78 (1.22–2.60) ^a^	0.003**	1.70 (1.14–2.53)^a^	0.01*
Vegetables < 3 spoons/day	22.5	16.9–27.9	0.001**^a^	1.88 (1.28–2.77) ^a^	0.001**	1.53 (1.02–2.30)^a^	0.04*
Fruits < 2 pieces/day	16.7	13.3–20.1	*0.33*^a^	1.20 (0.83–1.73) ^a^	*0.33*		
Whole grain bread < 3 slices/day	17.8	13.3–22.1	*0.23*^a^	1.26 (0.87–1.84)^a^	*0.23*		

* Statistical significance of *p* < 0.05

** Statistical significance of *p* < 0.005

^a^ The reference category is more than the indicated quantity of water, vegetables, fruits and bread respectively

The univariate analyses revealed that sex, age, water intake and vegetable intake had a significant influence on the likelihood of constipation, which was subsequently tested in a multivariate analysis (Table 2). There were no significant interactions between any of the variables used in the multivariate analysis. We found that in comparison to men, women were more than twice as likely to suffer from constipation (OR 2.08; 95% CI, 1.39–3.10). Moreover, the likelihood of constipation was significantly lower in the two older age groups than in the youngest age group of 18 to 38-year-olds (Table 2). Finally, the likelihood of constipation was significantly higher for the respondents with a low water and vegetable intake and a BMI classification of ‘obese’ (OR 1.70; 95% CI, 1.14–2.53, OR 1.53; 95% CI 1.02–2.30, and OR 1.72 95% CI, 1.02–2.90, respectively). We did not find a significant association between constipation and level of education and living environment.

**Fig. 1:**
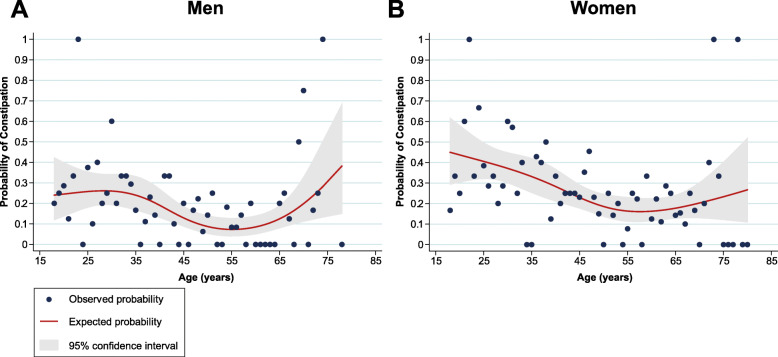
The probability of constipation in males and females plotted against the age of the respondents. The probability of constipation in males gradually decreased from 0.24 to a minimum value of approximately 0.08 at 56 years, after which the probability increased as respondents’ ages increased (**a**)**.** The probability of constipation in females showed a similar pattern, albeit with a higher starting value of 0.45 and with a more fluent decrease of probability down to a value of 0.17 at the age of 57 years, followed by a milder increase in probability as the age increased (**b**)

In addition, analysis of the probability of functional constipation for every year of age and different sexes showed that the probability varies at different age phases (Fig. 1). The lowest probability for both men and women is around an age of 55 years. Lastly, we found that obstetric history was not associated with an increased likelihood of constipation.

We performed a separate univariate analysis on the effect of obstetric history on the likelihood of constipation to further explore the differences between males and females. Following from this analysis we found that one or multiple vaginal deliveries were not associated with an increased likelihood of constipation in females (OR 0.72; 95% CI, 0.46–1.12). Furthermore, a history of difficulties with vaginal delivery, such as the use of an instrument or rupture and/or episiotomy, was also not associated with an increased likelihood of constipation (OR 0.72; 95% CI, 0.36–1.43).

### The pattern of constipation symptoms in the total study group

Next, we analyzed the prevalence of the symptoms included in the Rome IV criteria and other constipation symptoms among the sexes and in the different age groups of the total study group (Table 3). Of these, a low stool frequency was the only symptom that showed a comparable prevalence between the sexes. The other constipation symptoms were all reported significantly more often by women than by men. The 18 to 38-year-olds showed a significantly higher prevalence of all complaints than the older respondents, except for a hard or lumpy stool consistency, using the hands when defecating, and re-defecation within 1 h (Table 3).

**Table 3:** Constipation symptoms in the total study group

	Overall	Sex			Age			
	Total	Men	Women		18–38 years	39–54 years	55–80 years	
	n (%)	n (%)	n (%)	*p* value	n (%)	n (%)	n (%)	*p* value
**Total**	892 (100)	405 (100)	487 (100)		298 (100)	299 (100)	294 (100)	
***Constipation symptoms included in the Rome IV criteria for functional constipation***								
Straining ^a^	238 (26.7)	85 (21.0)	153 (31.4)	< 0.001**	110 (36.9)	70 (23.3)	58 (19.7)	< 0.001**
Incomplete defecation ^a^	191 (21.4)	63 (15.6)	128 (26.3)	< 0.001**	88 (29.5)	59 (19.7)	44 (15.0)	< 0.001**
Anal blockage ^a^	125 (14.0)	39 (9.6)	86 (17.7)	0.001**	58 (19.5)	38 (12.7)	29 (9.9)	0.002**
Hard or lumpy stool consistency	87 (9.8)	30 (7.4)	57 (11.7)	0.03*	32 (10.7)	26 (8.7)	29 (9.9)	*0.69*
Stool frequency less than 3 times a week	73 (8.2)	27 (6.7)	46 (9.4)	*0.13*	41 (13.8)	19 (6.3)	13 (4.4)	< 0.001**
Using the hands during defecation ^a,b^	18 (2.0)	4 (1.0)	14 (2.9)	0.05*	5 (1.7)	5 (1.7)	8 (2.7)	*0.58*
***Other constipation symptoms***								
Daily failure to defecate	126 (14.1)	38 (9.4)	88 (18.1)	< 0.001**	65 (21.8)	40 (13.3)	21 (7.1)	< 0.001**
Average straining duration of more than 5 min	132 (14.8)	48 (11.9)	84 (17.2)	0.02*	69 (23.2)	38 (12.7)	25 (8.5)	< 0.001**
Abdominal bloating	324 (36.3)	120 (29.6)	204 (41.9)	< 0.001**	142 (47.7)	119 (39.7)	63 (21.4)	< 0.001**
Anal pain ^a^	109 (12.2)	36 (8.9)	73 (15.0)	0.006*	62 (20.8)	26 (8.7)	21 (7.1)	< 0.001**
Abdominal pain ^a^	173 (19.4)	46 (11.4)	127 (26.1)	< 0.001**	83 (27.9)	63 (21.0)	27 (9.2)	< 0.001**
Re-defecation within 1 h of stool passage ^a^	159 (17.8)	59 (14.6)	100 (20.5)	0.02*	59 (19.8)	57 (19.0)	43 (14.6)	*0.21*

^a^ Complaints had to occur at least several times per month

^b^ Applying abdominal pressure with hands, manipulating the perineum, or removing stool from the rectoanal cavity with the fingers

* Statistical significance of *p* < 0.05

** Statistical significance of *p* < 0.005

### The pattern of constipation symptoms in the constipated respondents

**Fig. 2:**
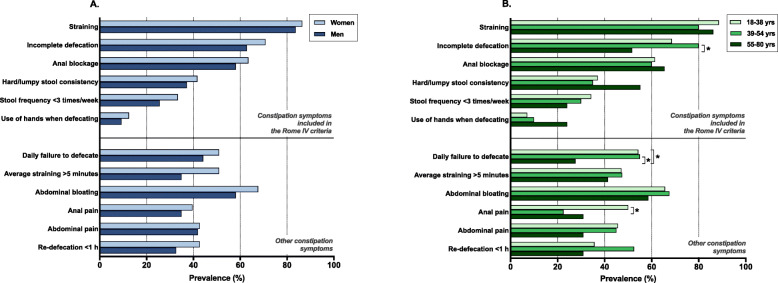
Constipation symptoms in the constipated respondents. No significant difference exists between men and women in the prevalence of any of the constipation symptoms (**a**)**.** Bonferroni correction of the comparison of constipation symptoms in three age groups shows significantly different prevalences of incomplete defecation between the middle and oldest age group (*p* = 0.038), of daily failure to defecate between the youngest and the oldest and the middle and the oldest age group (*p* = 0.046 and *p* = 0.073, respectively), and of anal pain between the youngest and middle age group (*p* = 0.012) (**b**)

We performed the same analysis of constipation symptoms in the subgroup of constipated respondents (*n* = 139). We found no significant differences between any of the investigated constipation symptoms in constipated men and women (Fig. 2). The comparison of the constipation symptoms between the three age groups showed significant different prevalences of incomplete defecation (*p* = 0.045), daily failure to defecate (*p* = 0.04), and anal pain (*p* = 0.011). Besides, Fig. 2b shows that some symptoms became less prevalent over time while others, for instance, a hard stool consistency, anal blockage, and using the hands when defecating are reported more frequently in the oldest age group.

### The pattern of constipation symptoms in constipated versus non-constipated respondents

**Fig. 3:**
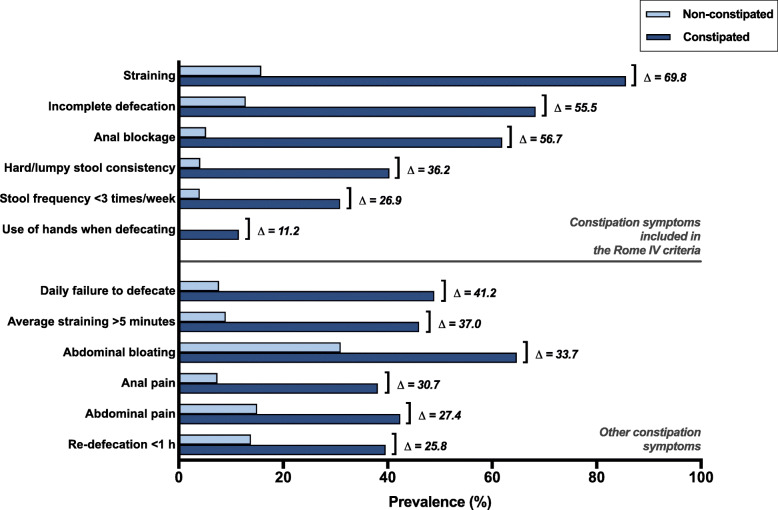
Constipation symptoms in constipated versus non-constipated respondents. The prevalences of all constipation symptoms were significantly different between the constipated and the non-constipated group (*p* < 0.001 for all symptoms). The highest differences in prevalence were found for the symptoms straining, incomplete defecation, and anal blockage

Finally, we compared the prevalence of all constipation symptoms between the constipated and the non-constipated respondents (Fig. 3). The prevalences of all the investigated symptoms were significantly different between the constipated and the non-constipated group (*p* < 0.001 for all symptoms). The most striking differences were found in case of straining (85.6% versus 15.8%, ∆ = 69.8%), incomplete defecation (68.3% versus 12.8%, ∆ = 55.5%), and anal blockage (61.9% versus 5.2%, ∆ = 56.7%). Not only the symptoms included in the Rome IV criteria of functional constipation, however, showed extensive differences in prevalence between the constipated and non-constipated respondents. We also found this for the other constipation symptoms. Especially daily failure to defecate (48.9% versus 7.7%, ∆ = 41.2%), average straining duration of more than 5 min (46.0% versus 9.0%, ∆ = 37.0%), and abdominal bloating (64.7% versus 31.0%, ∆ = 33.7%) showed marked differences in prevalence between the constipated and the non-constipated respondents. Notably, the differences in prevalence of a lumpy or hard stool consistency and a low stool frequency among constipated versus non-constipated respondents were comparable with the constipation symptoms that are not included in the Rome IV criteria.

## Discussion

This study demonstrates that not only the prevalence and likelihood of constipation, but also the clinical picture of constipated individuals varies according to certain demographic characteristics. Secondly, this study emphasizes that certain constipation symptoms that are not standard clinical practice in the diagnosis of constipation are, nevertheless, reliable indicators of constipation.

We found an overall prevalence of constipation of 15.6% in the Dutch population, which is in accordance with prevalences reported for other Western populations [2, 4, 6–9, 24]. To avoid a possible bias towards bowel functioning, we excluded respondents with relevant medical histories and/or who used medication known to have constipation as a side-effect. Viewing the prevalence of constipation from this perspective, 15.6% is remarkably high. Seeing that we used the Rome IV criteria to define constipation, this prevalence may differ slightly compared to studies that used previous versions of these criteria.

Sex and age were found to influence the likelihood of constipation independently of each other. Like other researchers, we found that women were more than twice as likely to suffer from constipation than men. Various theories have been proposed to explain this phenomenon, for example a slower gut transit in women due to the changing levels of progesterone and estrogen [25–27], or damage to the pelvic floor in a women’s obstetric history [28–30]. However, in this study we found no influence of one or more vaginal deliveries with or without complications on the likelihood of constipation in females. In this study, however, we found no influence of women’s obstetric history on the likelihood of constipation. Therefore, the exact cause of the higher likelihood of constipation in women remains unclear. It is important to continue searching for possible factors that may indicate different subtypes of constipation in the sexes and which may imply a different diagnostic approach depending on an individual’s sex. Detailed population-based studies about specific sex differences in bowel habits are scarce, and no clinically meaningful differences have yet been found [31]. We compared the clinical pattern of a wide range of constipation symptoms between men and women in our population-based sample. Since the prevalence of constipation in women is higher than in men, it is not surprising that in an analysis of the whole study group women suffered from almost all constipation symptoms more often than men. Remarkably, a comparison between women and men from only the constipated subgroup showed no significant differences in the prevalence of any of the constipation symptoms between men and women. Based on these two findings, we do not expect that in the general population men and women experience different subtypes of constipation, as the pattern of their reported symptoms is similar. The higher prevalence of symptoms in women could result from the fact that women have a higher tendency to report their physical symptoms [32], or from different central processing of rectal distension in women compared to men [27]. Future research in this field is still needed.

Age is another demographic variable that influenced the likelihood of constipation, with the youngest group more likely to suffer from constipation than older individuals. Our finding that the prevalence of constipation is highest at the younger ages agrees with existing literature. Nevertheless, it has also been reported that constipation is more prevalent in the elderly [1, 2, 4, 5, 7, 31]. These contradictory conclusions may be caused primarily by forming the age groups differently. The reasons behind the varying likelihood of constipation at different ages remain unclear. Therefore, we analyzed the clinical pattern of constipation symptoms in different age groups. We found that the prevalence of all constipation symptoms was higher in the 18 to 38-year-olds. When we specifically analyzed the prevalence of the symptoms among only the constipated individuals of the different age groups, we noted that prevalence fluctuated over time. Most symptoms became less prevalent with age, whereas symptoms like a hard stool consistency, anal blockage, and using the hands during defecation were reported more frequently in the oldest age group. This different clinical pattern might, for instance, be caused by an increased amount of neuropathy and/or pelvic floor muscle atrophy in the elderly, resulting in less propulsive intestinal movements and hard stool consistency, combined with a lower ability to expel stool. Taken together, we observed different clinical patterns of constipation symptoms depending on respondents’ ages. Future research is needed to determine the pathophysiological background of these observations.

In order to provide the clinician with more effective diagnostics for functional constipation, we compared the pattern of constipation symptoms between constipated and non-constipated individuals. In view of the fact that we diagnosed constipation in accordance with the Rome IV criteria [17], it is not surprising that straining, incomplete defecation, and anal blockage showed the most marked differences in prevalence. Remarkably, in addition to the Rome IV symptoms, constipated individuals also experience a broad spectrum of other constipation symptoms more frequently than non-constipated individuals. As already mentioned, deviating stool frequency and consistency are often considered the most obvious symptoms of constipation. Nevertheless, these two symptoms were reported with the same frequency as the constipation symptoms that are not included in the Rome IV criteria [17].

To improve the effectiveness of diagnosing constipation we advocate a more comprehensive clinical examination that includes other constipation symptoms. Our results imply that even individuals who do not fulfill at least two symptoms included in the Rome IV criteria, but who experience other constipation symptoms, could still suffer from functional constipation. This leads us to make the following recommendation: rather than limiting the clinical examination of individuals suspected of constipation to their compliance with the Rome IV criteria, extend the examination by obtaining additional information on such symptoms as daily failure to defecate and an average straining duration of more than 5 min.

A limitation of this study is that the data stemmed from an online survey. As a consequence, we may have missed a group of elderly people who are not active computer users. We may, therefore, have inadvertently selected the healthier individuals as representatives of the elderly group. This in turn would mean that the prevalence of constipation in the oldest group was in fact higher than reported. The advantage of an anonymous online survey is that it enabled us to obtain reliable information about an embarrassing topic. Another limitation is the lack of an objective assessment of constipation like for instance colonic transit time.

## Conclusions

We conclude that sex and age independently influence the likelihood of constipation in the general Dutch population. Moreover, differences between age groups in the clinical pattern of bowel complaints indicate the existence of different subtypes of constipation dependent on age. Our study highlights the need to examine individuals who might suffer from constipation more comprehensively. Clinicians should also enquire about constipation symptoms that are not included in the Rome IV criteria of functional constipation, for instance, daily failure to defecate and an average straining duration of more than 5 min. We encourage clinicians to adopt a more individual approach to the diagnosis of constipation. In our opinion this could lead to more effective treatment and better outcomes.

## Data Availability

The datasets used and/or analyzed during the current study are available from dr. P.M.A. Broens on reasonable request.

## Abbreviations

BMI: Body mass index
